# Low fermentable oligosaccharides, disaccharides, monosaccharides and polyols (FODMAP) diet improves symptoms in adults suffering from irritable bowel syndrome (IBS) compared to standard IBS diet: A meta-analysis of clinical studies

**DOI:** 10.1371/journal.pone.0182942

**Published:** 2017-08-14

**Authors:** Péter Varjú, Nelli Farkas, Péter Hegyi, András Garami, Imre Szabó, Anita Illés, Margit Solymár, Áron Vincze, Márta Balaskó, Gabriella Pár, Judit Bajor, Ákos Szűcs, Orsolya Huszár, Dániel Pécsi, József Czimmer

**Affiliations:** 1 Institute for Translational Medicine, Medical School, University of Pécs, Pécs, Hungary; 2 Szentágothai Research Centre, University of Pécs, Pécs, Hungary; 3 Institute of Bioanalysis, University of Pécs, Pécs, Hungary; 4 Department of Gastroenterology, First Department of Medicine, University of Pécs, Pécs, Hungary; 5 Hungarian Academy of Sciences - University of Szeged, Momentum Gastroenterology Multidisciplinary Research Group, Szeged, Hungary; 6 First Department of Surgery, Semmelweis University, Budapest, Hungary; Charité-Universitätsmedizin Berlin, Campus Benjamin Franklin, GERMANY

## Abstract

**Background:**

Irritable bowel syndrome (IBS) and functional digestive tract disorders, e.g. functional bloating, carbohydrate maldigestion and intolerances, are very common disorders frequently causing significant symptoms that challenge health care systems. A low Fermentable Oligosaccharides, Disaccharides, Monosaccharides and Polyols (FODMAP) diet is one of the possible therapeutic approaches for decreasing abdominal symptoms and improving quality of life.

**Objectives:**

We aimed to meta-analyze data on the therapeutic effect of a low-FODMAP diet on symptoms of IBS and quality of life and compare its effectiveness to a regular, standard IBS diet with high FODMAP content, using a common scoring system, the IBS Symptom Severity Score (IBS-SSS).

**Methods:**

A systematic literature search was conducted in PubMed, EMBASE and the Cochrane Library as well as in the references in a recent meta-analysis. Adult patients diagnosed with IBS according to the Rome II, Rome III, Rome IV or NICE criteria were included in the analysis.

**Statistical methods:**

Mean differences with 95% confidence intervals were calculated from studies that contained means, standard deviation (SD) or mean differences and SD of differences and p-values. A random effect model was used because of the heterogeneity (Q test (χ2) and I^2^ indicator). A p-value of less than 0.05 was chosen to indicate a significant difference.

**Results:**

The literature search yielded 902 publications, but only 10 were eligible for our meta-analysis. Both regular and low-FODMAP diets proved to be effective in IBS, but post-diet IBS-SSS values were significantly lower (p = 0.002) in the low-FODMAP group. The low-FODMAP diet showed a correlation with the improvement of general symptoms (by IBS-SSS) in patients with IBS.

**Conclusions:**

This meta-analysis provides high-grade evidence of an improved general symptom score among patients with irritable bowel syndrome who have maintained a low-FODMAP diet compared to those on a traditional IBS diet, therefore showing its superiority to regular IBS dietary therapy. These data suggest that a low-FODMAP diet with dietitian control can be a candidate for first-line therapeutic modality in IBS. Because of a lack of data, well-planned randomized controlled studies are needed to ascertain the correlation between improvement of separate key IBS symptoms and the effect of a low-FODMAP diet.

## Introduction

Irritable bowel syndrome (IBS) is a common chronic functional gastrointestinal disorder, which can be defined by the Rome IV criteria [1, 2]. IBS causes abdominal pain or discomfort, bloating and altered bowel habits (diarrhoea, constipation or a combination of these), without any pathological abnormality of the intestinal wall [3]. It can lead to significant impairment of quality of life (e.g. social isolation or stigmatization [4, 5]), decreased work productivity and an increase of health care and societal costs [6–9]. The incidence of the disease is high in Western countries, affecting 10–20% of the adult population, and it is twice more common among women [10, 11]. The exact pathomechanism remains unclear, but visceral hypersensitivity, altered gastrointestinal motility, changes in gut microbiota, altered brain–gut axis, low-grade digestive tract inflammation and psychological factors may play a role [12–14]. Because of the uncertain aetiology and pathophysiology, only a few effective, non-specific treatment options exist, improving only some key symptoms but not leading to the healing of IBS (laxatives, antidiarrhoeal agents, antispasmodics, antidepressants, and dietary and psychiatric interventions) [8, 15, 16]. Treatment is often multimodal, comprising dietary, psychological and pharmacological methods [15]. Several studies have proven that certain foods worsen the symptoms in most IBS patients because they play an important role in the development of those symptoms [17–23]. The most commonly reported foods are those containing lactose (milk, ice cream and yogurt) or fructose (honey, dates, oranges, cherries, apples and pears), gas-producing foods (beans, peas, broccoli, cabbage and bran), wheat and wheat-containing products, and sweeteners (sorbitol, mannitol and xylitol) [18]. These findings suggest that dietary intervention that excludes symptom-triggering food components could be a promising treatment option for IBS. Standard dietary interventions are detailed in some guidelines, e.g. the British Dietary Association and NICE guidelines [24, 25]. They recommend that patients regularly eat three meals and three snacks a day, never too much or too little, eat in peace and quiet, chew thoroughly, avoid certain foods (e.g. fatty or spicy foods, alcohol, coffee, onions, cabbage, beans, carbonated beverages, etc.) and eat fibre but distribute its intake over the day. A suggested main dietary approach is increased daily fibre intake; however, while improving general IBS symptoms in some subgroups, it can worsen them in others [26–29]. Reduction of dietary fat intake improved symptoms in patients because fatty acids can trigger symptoms in IBS [20, 23]. The effect of a gluten-exclusion diet is also controversial [30, 31]. A novel treatment option is a diet low in FODMAPs (Fermentable Oligosaccharides, Disaccharides, Monosaccharides and Polyols). Many popular, healthy foods have a high-FODMAP content, such as fruits (apples, pears, peaches and watermelons), vegetables (onions, garlic, squash and mushrooms), dairy products, grains (wheat and rye), and sweeteners (sorbitol and mannitol), etc. [32]. FODMAPs can trigger symptoms in IBS patients, based on two major mechanisms [8, 33–37]. The ‘small bowel hypothesis’ states that FODMAPs are unabsorbed, osmotically active molecules (carbohydrates), so they increase the intraluminal water content in the small intestine. This leads to distension, which causes symptoms such as bloating and discomfort. The increased distension also leads to faster oro-cecal transit, which impairs absorption in the small bowel [36]. The second mechanism (‘large bowel hypothesis’) describes FODMAPs reaching the colon unabsorbed, where they are rapidly fermented by colonic bacteria. This causes flatulence, bloating and discomfort through increased gas production and distension of the colonic wall [36]. Because of visceral hypersensitivity, the same magnitude of distension will produce different degrees of symptoms, depending on individual susceptibility [38]. These findings suggested that the exclusion of FODMAPs from the diet could improve IBS symptoms. A growing number of studies have shown a positive effect of FODMAPs on IBS symptoms. The need has thus arisen for a meta-analysis with a focus on effectiveness to provide evidence and underpin recommendations for wider therapeutic use. Our aim was to carry out a meta-analysis to prove whether a low-FODMAP diet improves the symptoms of adult IBS patients more effectively than other (standard) dietary interventions (i.e. without restriction of FODMAP content) recommended by the latest guidelines. Following the PRISMA 2009 guidelines, we used the PICO format to formulate our question (P: patients with IBS; I: low-FODMAP diet; C: high-FODMAP/standard IBS diet; O: IBS Symptom Severity Score (IBS-SSS)).

## Methods

### Search for articles

Our work was planned according to the PRISMA 2009 statement (S1 File). A systematic literature search was conducted by two independent reviewers (JC and PV) to find relevant articles on the effect of low-FODMAP dietary intervention in IBS up to 19 September 2016. The search covered three databases (PubMed, EMBASE and the Cochrane Library) with the terms ‘FODMAP AND irritable bowel syndrome’. For better targeting of synonymous phrases, we used the search terms: ‘FODMAP’ OR ‘FODMAPS’ OR ‘Fermentable poorly absorbed short chain carbohydrates’ OR ‘Fermentable oligosaccharides disaccharides monosaccharides and polyols’ as was done in a recent meta-analysis by Marsh et al. [39]. After this search process, language (only English) and species (only humans) filters were used and a title, and abstract screening was performed by the reviewers to identify potentially eligible articles. Disagreements were resolved by discussion. Duplicates were removed.

### Study selection

We included randomized controlled trials (RCT), non-randomized controlled trials and non-controlled prospective trials in our meta-analysis. Retrospective studies were excluded. The length of follow-up was not a reason for either inclusion or exclusion. Only articles written in English and those examining the effect of a low-FODMAP diet in human IBS patients were included in the meta-analysis. By definition, adult IBS patients (18 years or above) had to be diagnosed according to the Rome II, Rome III, Rome IV or NICE criteria. We enrolled controlled studies which included adult IBS patients as a control group. In the control groups, IBS patients had to follow a standard IBS diet (according to the guidelines) with significantly higher FODMAP content than in the intervention (low-FODMAP) group. As a standard, validated output measure, we searched for studies reporting the IBS Symptom Severity Score (IBS-SSS). The measurement of the severity of individual symptoms among the studies showed great heterogeneity (e.g. Visual Analogue Scale, different types of Likert scale, etc.), so we used only the complex IBS-SSS in our analysis as an outcome measure. Articles examining the results of patients with an organic disease (for example, inflammatory bowel disease and IBD) with functional gastrointestinal symptoms, which are similar to IBS symptoms, were excluded from the analysis.

### Quality assessment of the individual studies

The quality of RCTs was assessed with the frequently used Jadad score [40], while non-randomized and non-controlled prospective studies were evaluated according to the Methodological Index for Non-Randomized Studies (MINORS) [41]. Both scores were evaluated by JC and PV. Any disagreements were resolved by consensus.

### Data extraction

At the end of the screening process, relevant data were independently extracted from studies by the two reviewers (JC and PV). These included: IBS-SSS as the main outcome parameter, study design (e.g. randomized controlled trials, non-randomized controlled trials, etc.), basic characteristics of the study population (age, percentage of females and IBS subtypes), length of follow-up, diagnostic criteria for IBS and the size of the low-FODMAP and control (high-FODMAP) groups. Extracted data were validated by five co-authors (AG, IS, GP, ÁV and ÁS).

### Outcome measure

#### IBS-Symptom Severity Score (IBS-SSS)

This score provides a measure of overall IBS severity. It was validated by Francis et al. [42] in 1997 and consists of five questions that measure abdominal pain severity, abdominal pain frequency, abdominal bloating, bowel habit dissatisfaction and interference with quality of life on a 100 mm visual analogue scale (VAS). Patients should rate every symptom with a score from 0–100, so the theoretical range is 0–500 mm, with higher scores indicating a more severe disease. A final score of less than 175 indicates mild IBS, 175–300 shows moderate IBS, and >300 points to severe IBS [39].

## Statistical analysis

Data analysis was conducted with the Comprehensive Meta-Analysis software (Version 3.0, Biostat Inc.). In the forest plot analysis, mean differences with 95% confidence intervals were calculated from studies that contained means, standard deviation (SD) or mean differences and SD of differences and p-values. In one study (Pedersen et al. [43]), where the results were expressed as median, minimum and maximum values, we converted the data using the Hozo method [44].

The studies we included in the meta-analysis indicated that there is a considerable heterogeneity (different clinical methods, diverse participants, etc.), so the random effects model was used according to the DerSimonian and Laird method [45]. Statistically, heterogeneity was tested by Q test (χ2) and I^2^ indicator [46]. I^2^ indicator and Q tests were performed to assess whether the heterogeneity observed among effect sizes could be attributed to random chance or if other factors may play a role. The similar effect of non-investigated variables such as food intolerances and functional digestive tract disorders other than IBS could also cause IBS-like symptoms. I^2^ statistics represent the percentage of effect size heterogeneity that cannot be explained by random chance, but by other factors noted above. If the Q test is significant, it implies that the heterogeneity among effect sizes reported in the studies selected is more diverse than could be explained by random error only. The Q test was considered significant when p < 0.1.

We used subgroup analysis, with a p-value of less than 0.05 indicating a significant difference to compare the differences in the IBS-SSS between the control and low-FODMAP diet groups. Results from the meta-analysis were displayed graphically using forest plots. The potential for “small study effects”, including publication bias, was examined by visual inspection of funnel plots, in which the standard error was plotted against the net change for each study. In both funnel plots, an asymmetry could be observed, which could be caused by the subgroups. In S1 Fig the subgroup analysis indicates some publication bias, but in S2 Fig the same analysis suggests no such bias.

## Results

### Searching results

**Fig 1:**
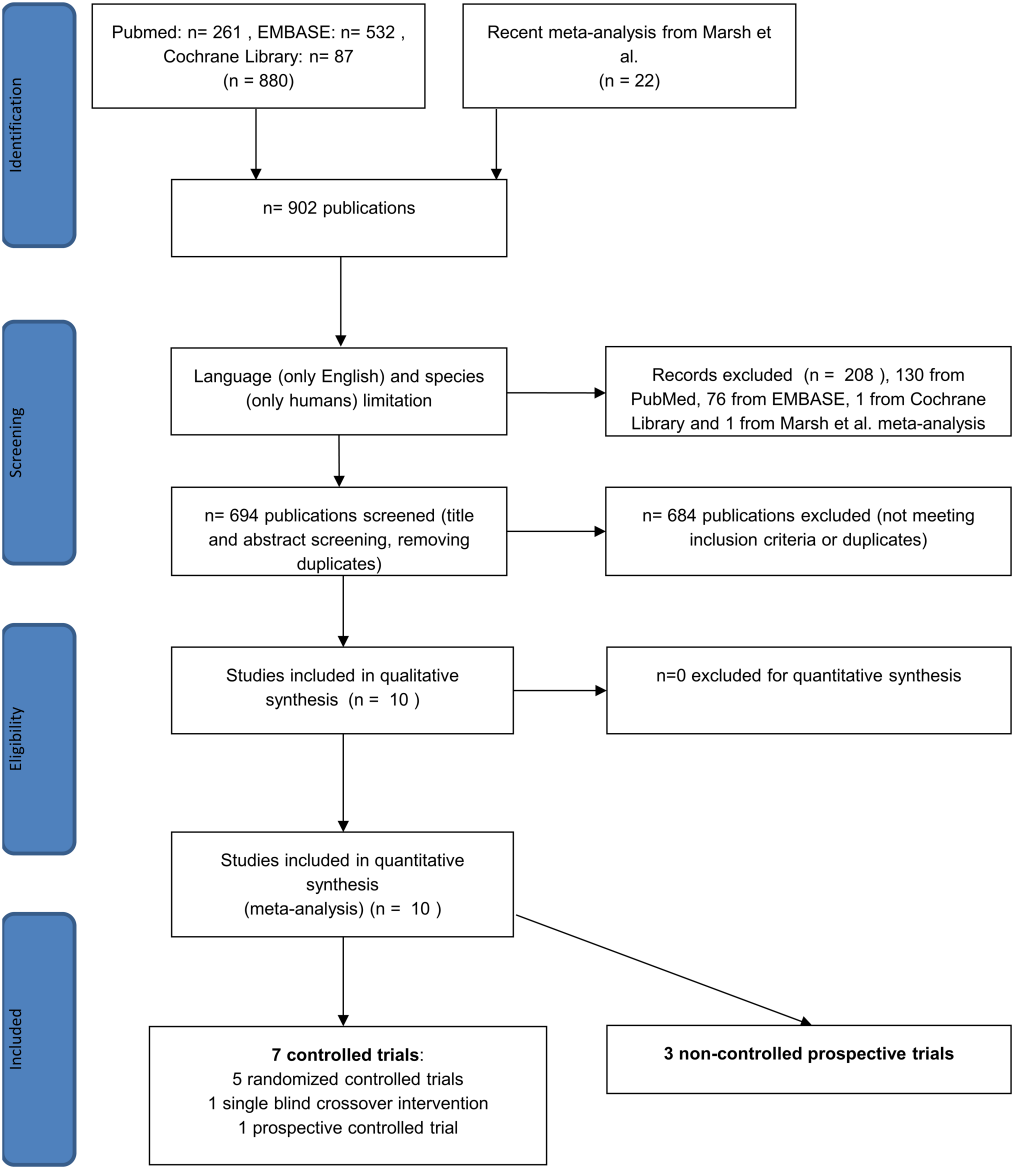
Flow chart for the systematic literature search.

Using the terms above, we found 880 articles in the three databases for evaluation, 261 in PubMed, 87 in the Cochrane Library and 532 in EMBASE. We also examined 22 further articles from the recent meta-analysis noted above [39], so 902 articles were found in total. After using the language (only English) and species (only humans) filters in EMBASE, PubMed and the Cochrane Library, 673 of 880 studies remained, and one of 22 was excluded from the meta-analysis by Marsh et al. [39] because it failed to meet the English-language inclusion criterion. After title and abstract screening and removing duplicates, 10 articles reporting on IBS-SSS eligible for further evaluation were found (Fig 1). Of these studies, 6 were available in full-text format, and 4 were short abstracts or supplements. The number of controlled trials was 7, and there were 3 non-controlled prospective studies. Of the controlled trials, 5 were randomized controlled trials (RCT), and 2 were non-randomized studies. At the time of the literature search, we found no eligible paper that used the most recent diagnostic criteria (Rome IV) for IBS. The basic characteristics of the articles are summarized in Table 1. The proportion of each IBS subtype in the studies included in the meta-analysis is detailed in Table 2. A quality assessment of the articles is summarized in Table 3.

**Table 1:** Baseline characteristics of the studies involved in the meta-analysis.

References	Country	Study design	Study duration	total or low-FODMAP/control cohort size	IBS diagnostic criteria	Age (years)	Percentage of females (%)
Böhn et al. [8]	Sweden	multi-centre, parallel, randomized, controlled, single-blind, comparative trial	4 weeks	33/34	Rome III	low-FODMAP: mean: 44 (18–69); control: mean: 41(18–68)	total: 81; low-FODMAP: 79; control: 84
McIntosh et al. [47]	Canada	prospective, randomized, single-blind, parallel study	3 weeks	18/19	Rome III	low- FODMAP: mean: 50.28 (26–77); control: mean: 5147 (24–83)	total: 86; low-FODMAP: 83; control: 89
Pedersen et al. (B) [16]	Denmark	randomized, non-blind, controlled trial	6 weeks	42/40 started, 34/37 finished	Rome III	low-FODMAP: median: 37 (18–71); control: median: 32 (18–73)	total: 77; low-FODMAP: 81; control: 72.5
Laatikainen et al. [48]	Finland	randomized, double-blind, 2x2 cross-over study	13 weeks (1- week run-in, 2x4- week intervention, 4 -week wash-out period)	80 started, 73 finished	Rome III	mean: 42.9 (21–64)	91
Schultz et al. (Suppl.) [49]	New -Zealand	randomized controlled trial	12 weeks	23/27	Rome III	no data	no data
Pedersen et al. (A) [43]	Denmark	single-blind, cross-over intervention	12 weeks (0–6 and 7–12 weeks)	19	Rome III	median 35 (18–74)	74
Piacentino et al. (Suppl.) [50]	Italy	prospective controlled trial	4 weeks	28/28	no data	21–68	68
Ones et al. (Suppl.) [51]	Norway	non-controlled prospective study	6 weeks	23	Rome III	mean: 35±11	87
Rossi et al. (Suppl.) [52]	Italy	non-controlled prospective study	8 weeks	12	Rome III	mean: 44.2±15.5	92
Valeur et al. [53]	Norway	non-controlled prospective study	4 weeks	63	Rome III	mean: 38.4 (19–67)	89

FODMAP = Fermentable Oligosaccharides, Disaccharides, Monosaccharides and Polyols; IBS = Irritable Bowel Syndrome; Suppl. = Supplementary material

**Table 2:** The proportion of each IBS subtype in the studies included in the meta-analysis.

References	IBS subtype	
	Low-FODMAP group (%)	Control diet group (%)
**Böhn et al.**	IBS-D: 26; IBS-C: 24; IBS-M/U: 50	IBS-D: 22; IBS-C: 35; IBS-M/U: 43
**McIntosh et al.**	IBS-D: 22; IBS-C: 6; IBS-M: 67; IBS-U: 5	IBS-D: 32; IBS-C: 5; IBS-M: 58; IBS-U: 5
**Pedersen et al. (B)**	IBS-D: 45; IBS-C: 12; IBS-M: 33; IBS-U: 10	IBS-D: 45; IBS-C: 17.5; IBS-M: 35; IBS-U: 2.5
**Laatikainen et al.**	IBS-D: 32.5; IBS-M: 62.5; IBS-U: 5	
**Schultz et al. (Suppl.)**	no data	no data
**Pedersen et al. (A)**	IBS-D: 42; IBS-C: 21; IBS-A: 37	
**Piacentino et al. (Suppl.)**	no data	no data
**Ones et al. (Suppl.)**	no data	-
**Rossi et al. (Suppl.)**	IBS-D: 25; IBS-C: 17; IBS-M: 58	-
**Valeur et al.**	IBS-D: 54; IBS-C: 16; IBS-M: 30	-

FODMAP = Fermentable Oligosaccharides, Disaccharides, Monosaccharides and Polyols; IBS = Irritable Bowel Syndrome; IBS-D = IBS-Diarrhoeal subtype; IBS-C = IBS-Constipation subtype; IBS-M/A = IBS-Mixed/Alternation subtype; IBS-U = IBS-Unsubtyped; Suppl. = Supplementary material. The number of patients in each IBS subtype groups is expressed in S1 Table.

**Table 3:** Quality assessment of the studies included in the meta-analysis.

	Study design	Jadad score	MINORS
**Böhn et al.**	multi-centre, parallel, randomized, controlled, single-blind, comparative trial	**3/5**	**-**
**McIntosh et al.**	prospective, randomized, single-blind, parallel study	**3/5**	**-**
**Pedersen et al. (B)**	Randomized, non-blind, controlled trial	**3/5**	**-**
**Laatikainen et al.**	randomized, double-blind, 2x2 cross-over study	**5/5**	**-**
**Schultz et al. (Suppl.)**	randomized controlled trial	**0/5**	**-**
**Pedersen et al. (A)**	single-blind cross-over intervention	**-**	**16/24**
**Piacentino et al. (Suppl.)**	prospective controlled trial	**-**	**15/24**
**Ones et al. (Suppl.)**	non-controlled prospective study	**-**	**8/16**
**Rossi et al. (Suppl.)**	non-controlled prospective study	**-**	**9/16**
**Valeur et al.**	non-controlled prospective study	**-**	**12/16**

Randomized controlled trials were evaluated with the Jadad score (0 = very poor, 5 = rigorous) [40]. Non-randomized studies were evaluated with the MINORS (Methodological Index for Non-Randomized Studies) [41], in which 12 items are scored (0: not reported; 1: reported, but inadequate; 2: reported and adequate). The global ideal score is 16 for non-comparative studies and 24 for comparative studies. Suppl. = Supplementary material.

### Low-FODMAP and control diets

Patients received dietary advice from a dietitian on a low- or high-FODMAP diet in 7 [8, 16, 43, 47, 49, 51, 53] of the 10 studies analyzed. 2 [50, 52] abstracts included in the meta-analysis failed to detail any information about dietitian involvement in the introduction of a low- or high-FODMAP diet. In a study by Laatikainen et al. [48], a special low-FODMAP diet containing rye bread was prepared for the patients, which had been developed and supplied by a bakery. Its FODMAP (fructan and mannitol) content was clearly lower than that of regular rye bread. The control group was not homogeneous among the studies, but it always had a significantly higher FODMAP content. The precise content of the foods used was only detailed in 2 trials [8, 48]; the others probably followed dietary guidelines. This uncertainty could have influenced our results.

### IBS-SSS

**Fig 2:**
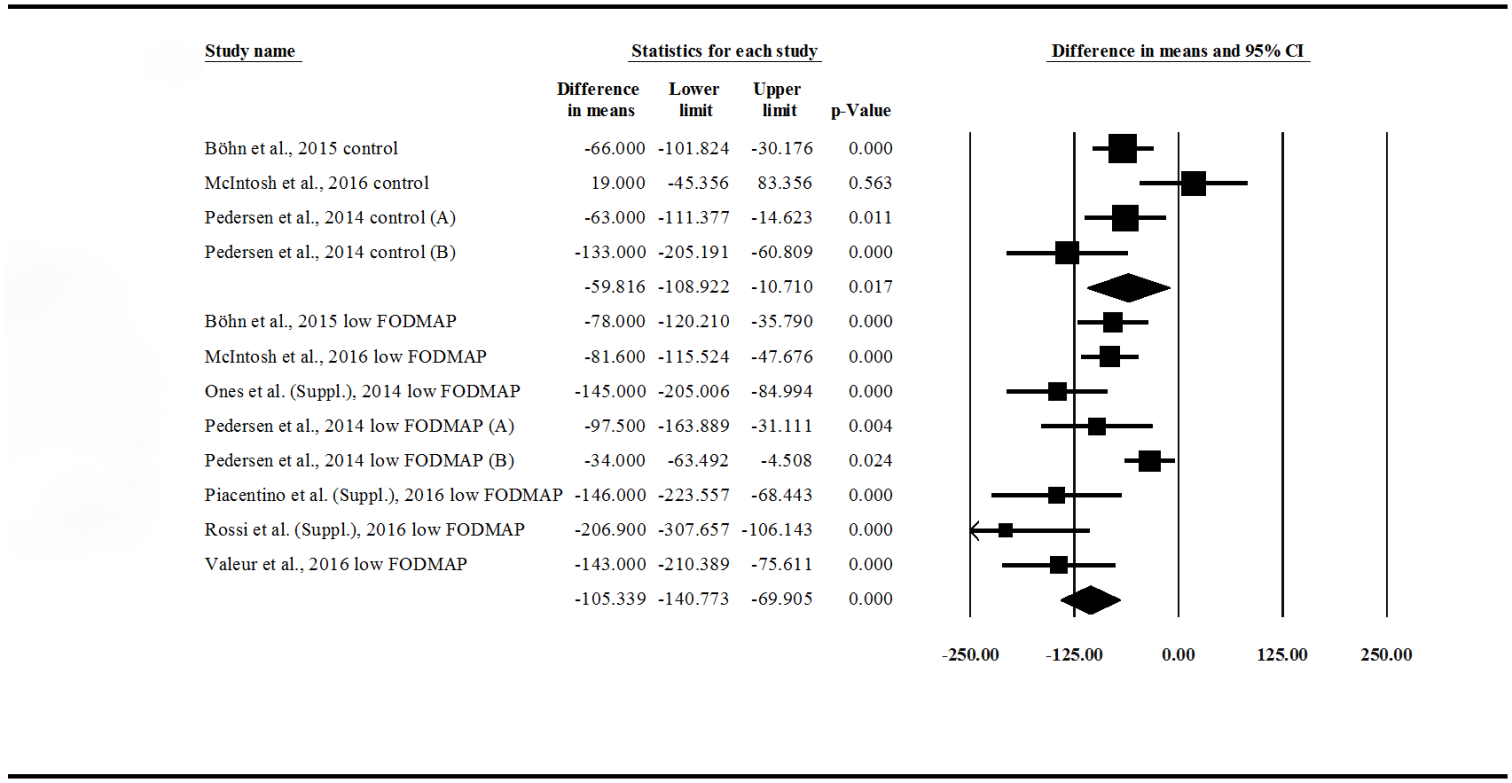
Forest plot of IBS-SSS DIMs, comparing pre- vs. post-intervention values within groups (low-FODMAP and control). IBS-SSS = Irritable Bowel Syndrome Symptom Severity Score (0–500); DIM = Difference in Means; FODMAP = Fermentable Oligosaccharides, Disaccharides, Monosaccharides and Polyols.

First, we wanted to see if a low-FODMAP diet is an effective treatment for irritable bowel syndrome. We compared the pre- vs. post-intervention IBS-SSS in control groups (4 publications) and low-FODMAP groups (8 publications) (Fig 2). There was a significant reduction in IBS-SSS in both control (difference in means (DIM), post- minus pre-values: –59.816 (95% CI: –108.922 ––10.710); p = 0.017) and low-FODMAP groups (DIM: –105.339 (95% CI: –140.773 ––69.905); p = 0.000). This means that both standard (high-FODMAP) and low-FODMAP diets are effective in improving symptoms and quality of life among IBS patients. The forest plot suggests that a low-FODMAP diet is more effective, but we cannot prove this statistically because of the overlapping confidence intervals. Significant heterogeneity was found between the studies: control group IBS-SSS values: Q = 9.837; df = 3; p = 0.02; I^2^ = 69.504%; low-FODMAP group IBS-SSS values: Q = 26.321; df = 7; p< 0.001; I^2^ = 73.405%.

**Fig 3:**
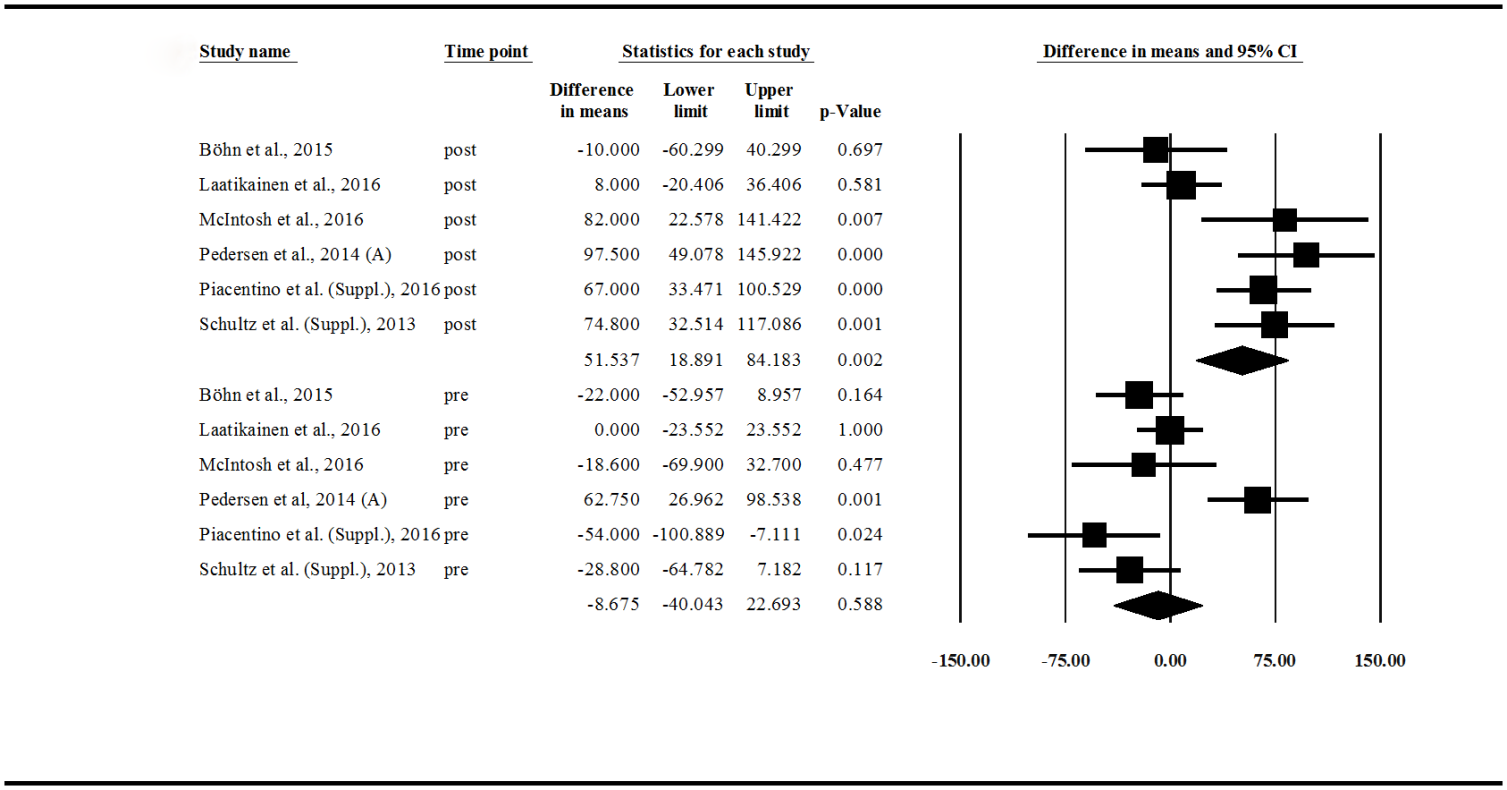
Forest plot of IBS-SSS DIMs, comparing pre- and post-intervention values between groups (low-FODMAP vs. control). IBS-SSS = Irritable Bowel Syndrome Symptom Severity Score (0–500); DIM = Difference in Means; FODMAP = Fermentable Oligosaccharides, Disaccharides, Monosaccharides and Polyols.

We compared (Fig 3) the pre- and post-intervention scores between the control and low-FODMAP groups in the controlled trials (6 publications for each group). This shows that there is no statistically significant difference in pre-values between the groups (DIM: control minus low-FODMAP values: –8.675 (95% CI: –40.043 –+22.693); p = 0.588), but a significant difference between post-values (DIM: +51.537 (95% CI: +18.891 –+84.183); p = 0.002) could be observed. These results confirm that the therapeutic effect of a low-FODMAP diet is better than standard dietary advice in patients with IBS. The meta-analysis also showed a significant heterogeneity: pre-IBS-SSS values: Q = 21.242; df = 5; p = 0.001; I^2^ = 76.462; post-IBS-SSS values: Q = 20.675; df = 5; p = 0.001; I^2^ = 75.816.

## Discussion

The standard dietary approach for IBS dietary therapy (e.g. high-fibre, low-fat, etc., as detailed above) recommended by guidelines only improves IBS symptoms to a limited extent. A growing number of recent studies have shown a beneficial effect of a low-FODMAP diet on IBS symptoms. Several of them have compared its efficacy to a standard IBS diet and challenged us to review the latest literature on the issue. A recent meta-analysis by Marsh et al. [39] analyzed the beneficial effect of a low-FODMAP diet on symptoms and quality of life in adult and pediatric patients with IBS and inflammatory bowel disease in the literature up to 24 March 2015. They only investigated the complex IBS-SSS only in 4 articles, and it was not stated whether low-FODMAP diet is significantly better than a control diet or not. We carried out our analysis on IBS-SSS, using more (10) articles, and we only focused on adult patients with IBS. A previous meta-analysis by Khan et al. and systematic review by Rao et al. [32, 54] also proved the efficacy of this diet on symptom improvement and suggested its introduction as a baseline treatment, but they could not state clearly whether it is better than standard dietary advice or not. Rao et al. [54] also investigated the high-fibre diet on chronic constipation and IBS. They performed a literature search up to September 2014 and did not conduct a statistical analysis due to heterogeneity and methodological quality. To our knowledge, this is the first meta-analysis to compare the effectiveness of low-FODMAP foods to a regular IBS diet recommended by the guidelines.

A pooled analysis of 7 controlled trials (5 randomized and 2 non-randomized) and 3 non-controlled trials confirmed that a low-FODMAP diet significantly improves general symptoms (IBS-SSS) in patients with IBS compared to standard dietary recommendations and a high-FODMAP diet. FODMAPs are poorly absorbed carbohydrates that cause an increase of water content in the bowel based on the osmotic effect and increased gas production by colonic bacterial flora. These effects of FODMAPs induce several symptoms in patients with irritable bowel syndrome and numerous patients with functional gastrointestinal disorders mainly by distension and the osmotic laxative effect [8, 36, 37].

The pooled sample size was large, and the expression of the data from the studies enrolled was acceptably homogeneous with regard to the key question of the meta-analysis.

Because of the considerable heterogeneity of the expressed data, the random effects model was used with the DerSimonian and Laird [45] method for analysis. This is possible because of the similar effect of non-investigated variables such as food intolerances and functional digestive tract disorders other than IBS that could cause IBS-like symptoms as well. It would be important to study the effect of a low-FODMAP diet in these groups to better understand the role of a food challenge in provoking uncompliant symptoms of functional digestive tract disorders, as in IBS. Nevertheless, the beneficial effects of a low-FODMAP diet on IBS-SSS were statistically significant even in the heterogeneous population analyzed, thus supporting the high impact of this diet on IBS symptoms.

Study data reflected some publication bias based on heterogeneity (S1 Fig) and no significant bias based on time-based comparisons of the low-FODMAP diet (S2 Fig).

We evaluated the quality of the studies included in the meta-analysis (Table 2, S2 Table) using the Jadad score for RCTs and MINORS for non-randomized studies. Among RCTs, the Schultz et al. trial [49] was an outlier (Jadad score = 0). This could be due to the fact that it is only available in abstract form; there is therefore a lack of information on the study design. The scores given to the other trials were satisfactory.

The strength of our study is that a standardized complex outcome score (IBS-SSS) was used to measure the therapeutic effect. This score measures abdominal pain frequency and severity, bloating, dissatisfaction with bowel habit and quality of life together on a 0–500 mm Visual Analogue Scale (VAS). This scoring system provides information not only about symptoms, but also about quality of life. A sufficient number of articles were found to carry out an accurate statistical analysis, using this important outcome score. With this work, we proved not only the positive effect of a low-FODMAP diet on IBS-SSS, but also its superiority to a high-FODMAP standard IBS diet. Our meta-analysis is the first to provide unambiguous, high-level evidence for the superiority of a low-FODMAP diet to a standard dietary approach in the improvement of general symptoms and well-being among patients with IBS. These data suggest that the first-line introduction of a low-FODMAP diet in the treatment of IBS could improve the therapeutic effect on IBS symptoms and might decrease health care-related and societal costs [9].

There are some limitations to our study. First, we focused on the complex IBS-SSS, and due to the lack of detailed published data, we did not perform a statistical analysis of the individual symptoms in the symptom score. Therefore, it is not clear which of the five elements play a key role in the improvement of IBS symptom severity toward better personalization of this dietary approach. The main reason was the lack of data and control groups, as well as the heterogeneity in the literature in measuring symptom severity (e.g. VAS and different types of Likert scale). A uniform, consensus-based, well-comparable measurement of symptom severity (e.g. IBS-SSS) is suggested for use in future studies. Second, we included not only full-text articles, but also 4 short supplements [49–52] in our analysis, thus increasing the quantity of data on control groups. Third, because of the lack of data in the different IBS subtypes, it is not clear which subgroup experienced the greatest symptom improvement. Finally, the standard IBS diet group was not homogeneous. The control diet always contained a significant number of FODMAPs; however, only 2 out of 10 studies detailed exact food contents [8, 48]. Others probably used IBS dietary guidelines; thereafter, some differences were realized between contents, thus potentially influencing our results.

To prove the effect of a low-FODMAP diet on bowel movement frequency in IBS patients and to demonstrate which IBS subgroup could profit significantly from this diet, more double-blind, randomized controlled trials should be conducted with the following standardized parameters. Only patients fulfilling the most recent diagnostic criteria for IBS (Rome IV) should be included in studies. A precise description of the contents of the diets studied is crucial for an accurate analysis. It is highly recommended dietitians be involved in guiding patients on diets to avoid significant differences within study groups and inadequate nutrient intake. Patients should also be adequately monitored during trials to ensure their adherence to a particular diet. Uniform outcome measures should be used to make studies scientifically comparable. Except for measuring symptom severity on the VAS scale only, it is suggested that a more detailed IBS-SSS be used in each subtype of the IBS patient group, which measures not only the severity of the main symptoms, but also the quality of life. Yao et al. [55] discuss the crucial points and difficulties of designing clinical trials in dietary interventions in patients with functional gastrointestinal disorders.

## Conclusion

This meta-analysis confirms that a diet low in fermentable oligosaccharides, disaccharides, monosaccharides and polyols (FODMAP) significantly improves general symptoms and quality of life in patients with irritable bowel syndrome. Our analysis of the appropriate literature data also confirms that a low-FODMAP diet is more effective than standard IBS dietary therapy in patients diagnosed with IBS. However, a low-FODMAP diet raises certain issues, such as the alteration of gut microbiota and inadequate nutrient intake without dietitian assistance. The possible health advantages of a low-FODMAP diet—when it is effective—compared to medical treatment require further evaluation. In consideration of its possible limitations and based on findings from this meta-analysis, a low-FODMAP diet could be a potential first-line and supplementary dietary therapeutic approach with the aid of a dietitian for patients with irritable bowel syndrome to improve abdominal discomfort, abdominal pain, bloating and quality of life. Because of the lack of published data, it is not possible to prove the effect of a low-FODMAP diet on bowel movement frequency in IBS patients. It also remains unclear which IBS subgroup could profit most from this diet. More randomized controlled trials are called for to analyze these effects of dietary approaches.

## Supporting information

S1 Table Raw data material. (XLSX)

S2 Table Jadad and MINOR scores of the articles included in the meta-analysis. (XLSX)

S1 Fig Funnel plot of publication biases among the studies in Fig 2. ES = effect size; s.e. of ES = standard error of effect size.
(TIF)

S2 Fig Funnel plot of publication biases among the studies in Fig 3. ES = effect size; s.e. of ES = standard error of effect size.
(TIF)

S1 File PRISMA 2009 checklist. (DOC)

## Abbreviations

CI: Confidence interval
DIM: Difference in means
ES: Effect Size
Fig: Figure
FODMAP: Fermentable Oligosaccharides, Disaccharides, Monosaccharides and Polyols
GI: Gastrointestinal
IBD: Inflammatory bowel disease
IBS: Irritable bowel syndrome
IBS-SSS: Irritable Bowel Syndrome Symptom Severity Score
MINORS: Methodological Index for Non-Randomized Studies
mm: millimetre
NICE: National Institute for Health and Care Excellence
PICO: Population/Problem, Intervention, Comparison, Outcome
PRISMA-P: Preferred Reporting Items for Systematic Review and Meta-Analysis Protocol
RCT: Randomized controlled trial
SD: Standard deviation
s.e.: Standard error
S/Suppl.: Supplementary material
VAS: Visual Analogue Scale
