# Supplementary figures and images for: Low fermentable oligosaccharides, disaccharides, monosaccharides and polyols (FODMAP) diet improves symptoms in adults suffering from irritable bowel syndrome (IBS) compared to standard IBS diet: A meta-analysis of clinical studies
Source: PLoS One. 2017 Aug 14;12(8):e0182942. doi: 10.1371/journal.pone.0182942 (PMC5555627; doi:10.1371/journal.pone.0182942)

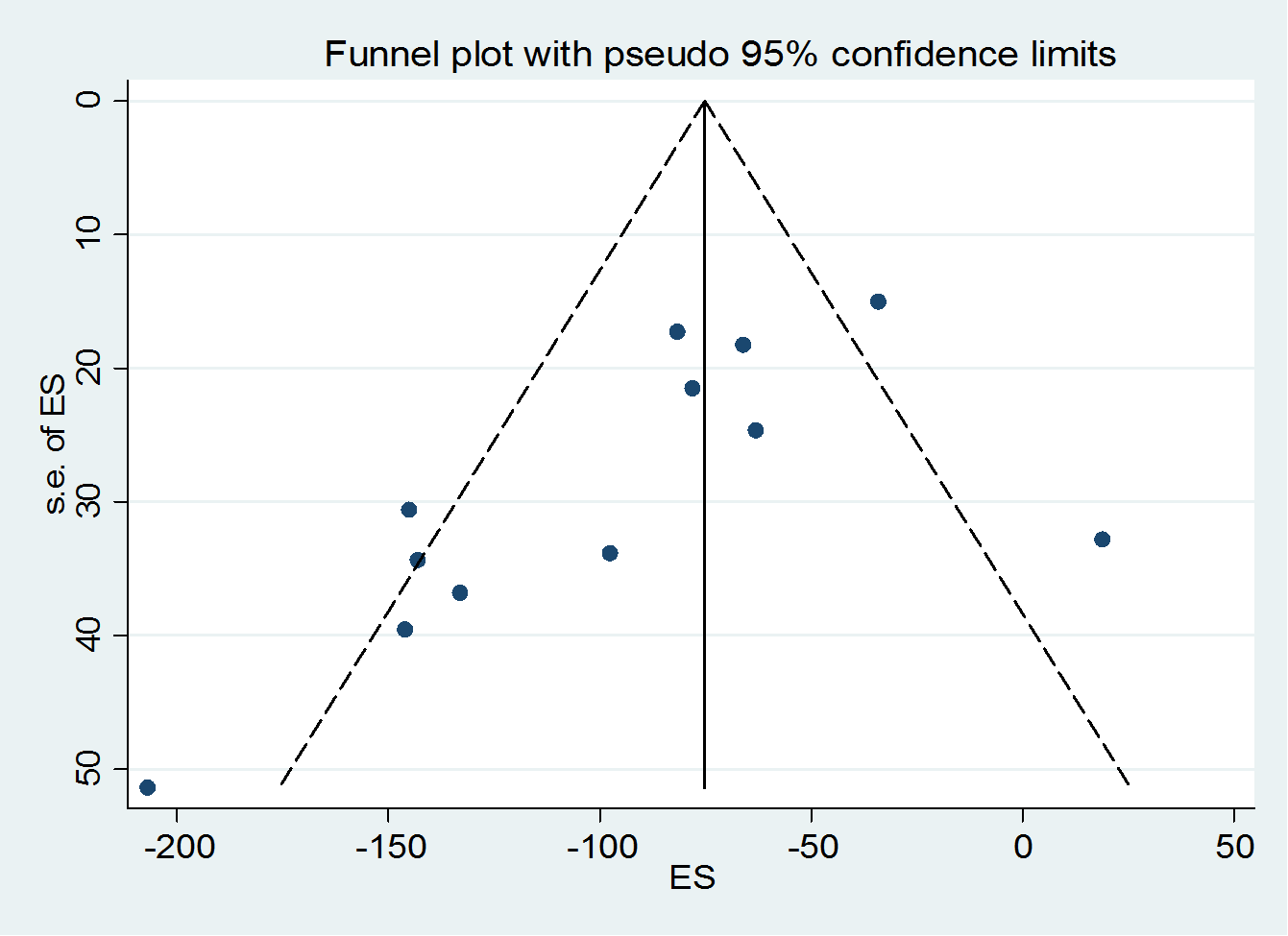

Supplement: S1 Fig — ES = effect size; s.e. of ES = standard error of effect size. (TIF) [file pone.0182942.s003.tif]

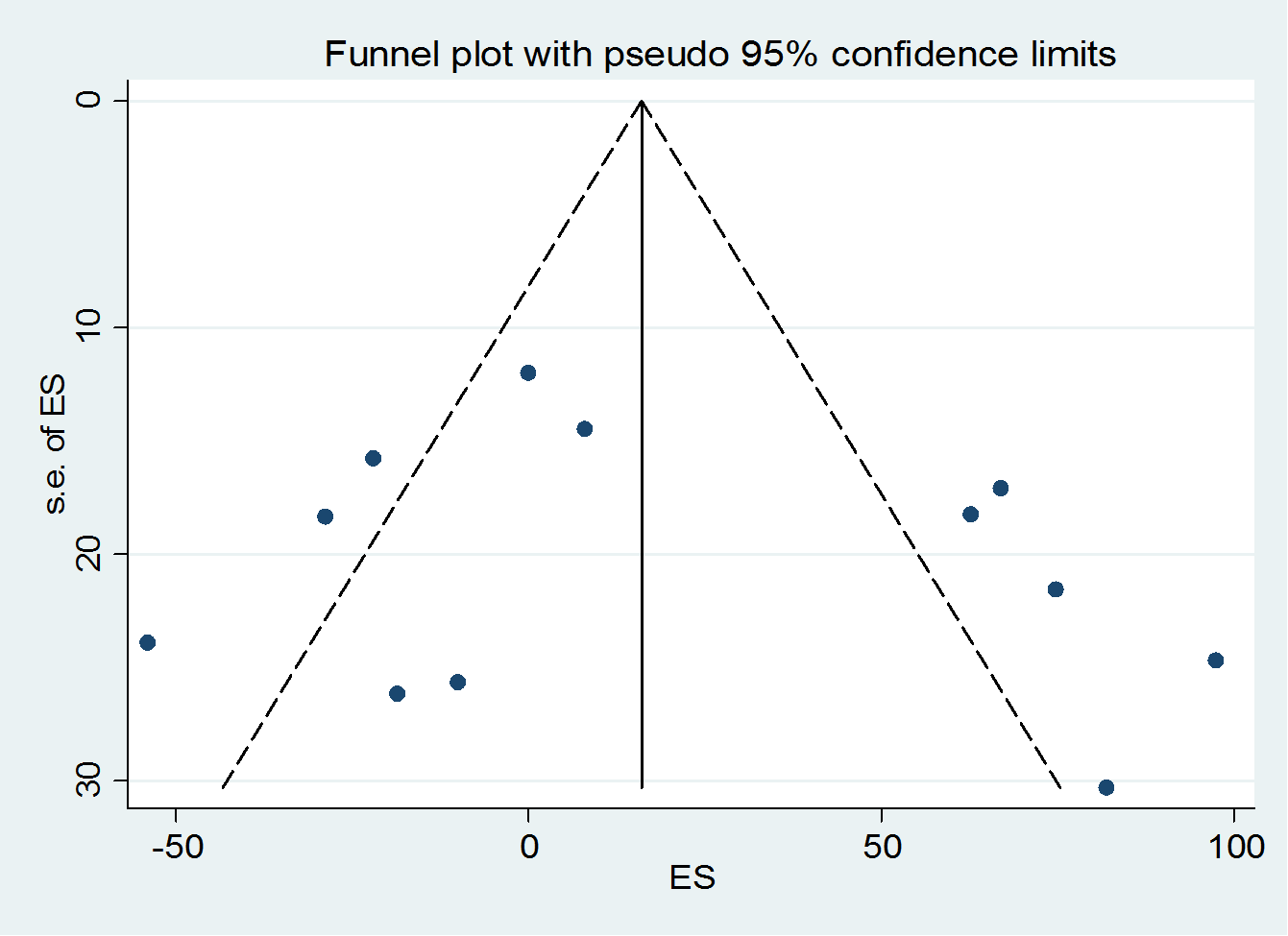

Supplement: S2 Fig — ES = effect size; s.e. of ES = standard error of effect size. (TIF) [file pone.0182942.s004.tif]
